# A novel hybrid framework integrating GA-driven 3D ResUNetGAN for MRI brain tumor segmentation

**DOI:** 10.1371/journal.pone.0349451

**Published:** 2026-05-18

**Authors:** Muthulakshmi Kirubakaran, Jayalakshmi Mohan

**Affiliations:** 1 School of Advanced Sciences, Vellore Institute of Technology, Vellore, Tamilnadu, India; 2 School of Computer Science and Engineering, Vellore Institute of Technology, Vellore, Tamil Nadu, India; The Hong Kong Polytechnic University, HONG KONG

## Abstract

Accurate brain tumor segmentation by multi-modal MRI is crucial for diagnosis, treatment planning, and prognostic assessment. This work proposes a novel hybrid architecture within a GA optimized by 3D residual U-Net (ResUNet) with generative adversarial network (GAN) for the accurate segmentation of tumor subregions, namely the tumor core (TC), whole tumor (WT), and enhancing tumor (ET), using a dataset collected from BraTS2023 (437 training and 188 validation). By using GAs, we enable the computer to automatically find the best model structure and training settings, which helps keep the model simple enough to avoid overfitting while being powerful enough to generalise well to new data. The GAN part of our method trains the model to draw more realistic and consistent boundaries around tumor regions in MRI scans, thereby improving the overall quality of the segmentation. In our experiments, this GA-ResUNetGAN approach performed better than several leading methods, such as 3D UNet, DynUNet, UNETR, SwinUNETR, and the standard ResUNet, especially in accurately identifying TC, WT, and ET parts—achieving Dice scores of 0.94, 0.96, and 0.91 on the validation set. In addition, K-Fold cross-validation was performed to ensure consistent performance across different data splits. While the method involves more processing work than some baseline techniques, it provides improved segmentation quality and boundary delineation on the evaluated dataset. These results demonstrate the advantages of combining evolutionary optimization with adversarial deep learning for challenging medical imaging tasks.

## Introduction

Brain tumors, especially malignant gliomas such as astrocytomas, are among the most aggressive and lethal neurological disorders, often leading to high mortality rates despite advances in treatment. Early and accurate identification is critical for improving patient outcomes, and magnetic resonance imaging (MRI) is particularly useful for detecting, assessing, and monitoring malignant tumors due to its high tissue contrast and non-invasive nature [1]. However, manually segmenting brain tumors from MRI data is time-consuming, subjective, and prone to inter-expert variability. MRI is an essential tool in brain tumor diagnosis, allowing for thorough imaging of tumor characteristics employing modalities such as T1n, T1c, T2w, and T2f. The brain tumor segmentation challenge, especially the BraTS dataset, provides a complete environment for academics to investigate automated segmentation techniques, allowing them to address tumor delineation issues [2].

In recent years, machine learning (ML) and deep learning (DL) methods have played an important role in medical image analysis by enabling automated and more accurate tumor segmentation. Traditional ML approaches, including support vector machines and random forests, have been applied to feature-based classification tasks. At the same time, DL approaches, especially Convolutional Neural Networks (CNNs) such as U-Net, are becoming better at capturing spatial and contextual features for segmentation [3,4]. Recent advances in multimodal learning using transformer-based architectures and contrastive representation learning have further improved the integration of heterogeneous biomedical data sources [5]. Havaei et al. (2017) developed a multi-scale CNN architecture for brain tumor segmentation, achieving competitive performance on the BraTS 2013 and 2015 datasets [6]. Kamnitsas et al. (2017) presented DeepMedic, a 3D convolutional neural network combined with conditional random fields to improve segmentation accuracy by better leveraging spatial context [7]. Because of its encoder–decoder structure and efficient multi-scale feature representation, the U-Net architecture, which was presented by Ronneberger et al. (2015), is extensively used in medical image segmentation [8]. By using residual connections that enhance training stability and mitigate vanishing gradient problems, ResUNet expands on this architecture [9]. Generative adversarial networks (GANs), first proposed by Goodfellow et al. (2014), have also been applied in medical imaging to generate realistic synthetic data and enhance segmentation results [10,11]. For example, Han et al. (2021) used a GAN-based approach to improve tumor boundary detection in MRI images, thereby increasing Dice scores on the BraTS dataset [12,13]. Optimization methods like Genetic Algorithms (GAs) [14] have been employed more often in combination with deep learning models to enhance network architectures and modify hyperparameters. Anaraki et al. (2025) showed that GAs may enhance CNN-based glioma classification, with an accuracy of 90.9% on MRI datasets [15]. By using evolutionary algorithms to modify network parameters, Wang et al. (2023) improved segmentation performance [16]. A number of studies have examined hybrid approaches to improve segmentation resilience in a variety of data scenarios, such as combining convolutional neural networks with attention mechanisms or transfer learning [17,18].

Despite these advancements, a number of issues still exist, most notably data imbalance, noise sensitivity, and the high computing requirements of high-resolution 3D MRI data [19]. These problems have been addressed by recent research using sophisticated loss functions [20], multi-modal data fusion [21], and data augmentation strategies [22]. For instance, Isensee et al. (2021) suggested a modified U-Net design for the BraTS challenge that enhanced performance by meticulous preprocessing and architectural improvement [23]. A transformer-based segmentation method that uses self-supervised learning to identify long-range correlations was also presented by Zhou et al. (2022) [24].

This paper introduces a hybrid, optimization-driven framework for brain tumor segmentation on the BraTS2023 dataset, building on existing challenges and prior research efforts. The suggested method combines GAs with a ResUNet-based GAN architecture, where the GAN component improves segmentation by producing high-quality tumor masks and residual connections improve feature extraction. In order to solve problems with overfitting and data variability, GAs are used to improve network designs and hyperparameters. A reliable baseline for comparing the suggested method to other strategies is the BraTS2023 dataset, which consists of multi-modal MRI images with expert-annotated ground truth. This work attempts to provide better tumor border delineation, increase resilience across MRI modalities, and improve segmentation accuracy using evolutionary optimization. The proposed framework contributes to the growing body of hybrid ML–DL methods that support scalable and accurate brain tumor segmentation for clinical decision-making. The main contributions of this work are summarized as follows:

- The model can learn both voxel-wise accuracy and global structural consistency in clinical image segmentation by integrating a GAN framework with a 3D ResUNet-based generator and a 3D convolutional parser.
- Three therapeutically relevant tumor subregions—tumor core (TC), whole tumor (WT), and enhancing tumor (ET)—are created from the original BraTS labeling using a special transformation procedure. Precise per-class segmentation estimates are made possible by the model’s simultaneous training of many channels.
- The hyperparameters and network configurations of the ResUNetGAN model are tuned using GAs that take into account initial filter sizes, learning rates, and negative loss weights. This evolutionary optimization addresses issues such as overfitting and data variability, thereby improving model robustness and performance.
- To evaluate performance across three tumor subregions, the framework incorporates quantitative evaluations using DSC, precision, sensitivity, specificity, F2 score, PSNR, MAE, and HD95. Additionally, the predicted masks are superimposed on MR slices using slice-wise visualization to facilitate qualitative assessment.

**Fig 1:**
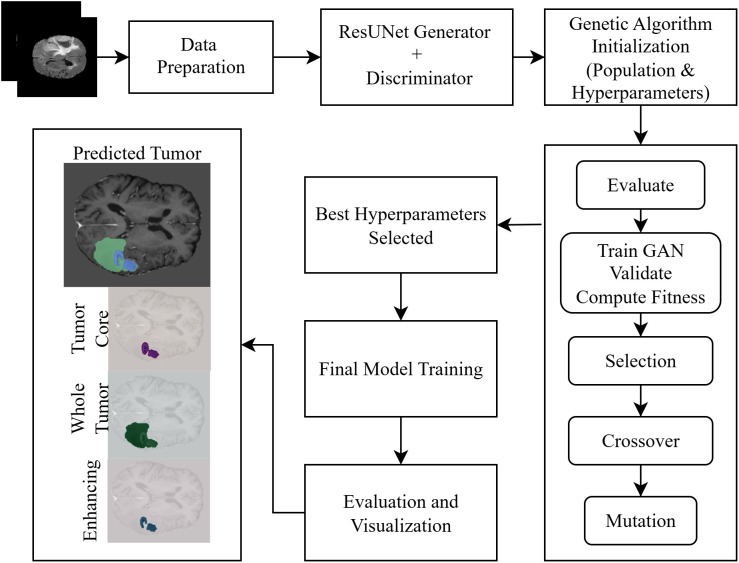
Scheme of the proposed model.

Fig 1 illustrates the overall workflow of the proposed GA-ResUNetGAN framework, including preprocessing, optimization, and segmentation stages.

This article follows the following structure: The **Research Motivation** section discusses the motivation for this research and highlights existing limitations in the field. The suggested hybrid GA-ResUNetGAN approach is presented in the **Methodology** section, along with information on evaluation criteria and data preparation. The main conclusions and experimental data are presented in the **Experimental Results** section. A thorough discussion and comparison with previous research are given in the Discussion section. Finally, the **Conclusion** section summarizes the main conclusions and outlines directions for future research.

## Research motivation

Brain tumor segmentation remains one of the most difficult challenges in medical imaging. This is owing to the brain’s complex structure, the variety of tumor forms, intensity heterogeneity, and the scarcity of high-quality annotated datasets. The Dense-Inception U-Net [25] was an early model that combined Inception-Residual blocks with dense connections within a U-Net architecture to extract multi-scale features. This increased the depth and spatial feature representation without significantly increasing the number of parameters. However, such models continue to struggle with data scarcity and require human customization of architecture and training settings.

To address insufficient training data, GANs were used to augment the datasets. TumorGANet [26] combined transfer learning with ResNet50 and GAN-based data augmentation. Moreover, several recent medical imaging studies have applied reinforcement-driven or swarm-based optimization schemes demonstrating their applicability in clinical learning environments. Examples include reinforcement–swarm learning for fMRI decoding [27], RL-inspired differentiable optimization frameworks for complex biological systems [28], chaotic–swarm fuzzy inference for multi-class neuro-imaging [29], tumor growth modelling using hybrid optimization frameworks [30], and fuzzy–swarm segmentation approaches in medical imaging [31]. These works collectively confirm that meta-heuristic intelligence can assist deep medical analysis, motivating the integration of GA within our proposed segmentation pipeline.

This led to considerable gains in categorization accuracy. Similarly, another GAN-based technique [32] combined autoencoders with DenseNet. It accelerates the speed of training and improves segmentation. However, these approaches continue to rely on fixed model configurations and often require human adjustments to hyperparameters for new data conditions.

Unpaired GAN models like RescueNet [33] addressed the annotation burden by using residual cyclic encoders to train on unlabeled data. They showed that adversarial learning can work without ground truth. The dual-discriminator approach in the Dual-Discriminator Conditional Generative Adversarial Network (DDCGAN) [34] enhanced feature extraction by using empirical wavelet transforms and a heuristic optimization method, Border Collie optimization (BCO). Although these methods have shown potential, the use of preset optimizers and rigid structures has hampered their ability to adapt to various datasets. Several studies have looked into enhancing deep learning models with GA to overcome these limitations. GA was used in studies [35,36] to improve CNN hyperparameters and architectures. This resulted in better performance and less overfitting. Research in [37–39] showed that combining GA with CNNs and deep networks led to better models. GA assisted in optimizing the weights and training dynamics. Comprehensive examinations, such as [40], have demonstrated GA’s utility in optimizing complex deep learning pipelines. Other meta-heuristic optimization strategies, such as DMVO-based approaches for constraint optimization, have also shown effectiveness in biologically inspired computational problems [41]. Interestingly, hybrid models such as GAN-GA [42] have demonstrated that GA can improve the learning of generative models, producing more realistic synthetic images.

Some approaches combined GA with U-Net architectures [43] for both segmentation and feature selection. Dual-GAN ensembles and custom loss functions [44,45] were used to increase robustness under data imbalance. GAN-based data augmentation [46] and multi-objective swarm optimizers [47] demonstrate the effectiveness of hybrid learning and optimization methods.

To contextualise the role of optimization in brain tumor analysis, Table 1 summarises recent meta-heuristic-based deep learning approaches, along with their advantages and limitations.

**Table 1:** Recent meta-heuristic optimization in deep learning approaches applied to brain tumor analysis.

Ref	Methodology	Results	Advantages	Disadvantages
[48]	CNN for brain tumor classification with meta–heuristic optimization of network parameters	High classification accuracy on MRI brain tumor datasets	Shows that meta–heuristic tuning improves CNN performance; relatively simple CNN–based pipeline	Focused on 2D classification; not detailed 3D subregion segmentation; optimizer not easily generalized
[49]	Automated CNN–based system for brain tumor diagnosis; meta–heuristic optimizer tunes CNN hyperparameters	Improved diagnostic and classification performance vs. non–optimized CNN baselines	Fully automated pipeline; demonstrates benefit of meta–heuristic–driven hyperparameter search	Primarily classification–oriented; segmentation of tumor subregions not addressed
[50]	EOA used with deep models for MRI brain tumor segmentation and classification	High segmentation and classification metrics on MRI datasets	Integrates meta–heuristic optimization directly with segmentation network; joint segmentation–classification framework	Multi–stage complex architecture; optimizer strongly coupled to model design
[51]	Brain tumor segmentation using optimized depthwise separable CNN combined with Dense U–Net	Improved Dice scores and parameter efficiency vs baseline U–Net	More efficient architecture; good trade–off between accuracy and complexity	Mainly 2D slice–wise segmentation; optimization focused mostly on architecture
[52]	Optimized U–Net–based framework for brain tumor segmentation and classification	Better segmentation and classification than standard U–Net	Confirms U–Net optimization enhances tumor analysis	Uses task–specific optimization; limited transferability to 3D multi–modal BraTS settings
[53]	Deep learning architecture for brain–tumor classification with improved Hunger Games Search meta–heuristic	High classification accuracy and improved convergence vs standard training	Demonstrates effectiveness of improved HGS meta–heuristic	Focused on classification; does not address voxel–level segmentation; optimization tuned to specific dataset
[54]	Enhanced brain–tumor segmentation using image registration and optical particle swarm optimization with ResNet–Inceptionv2 HCNN	Strong segmentation performance; improved registration–driven localization	Combines registration, deep features, and PSO–based optimization	Pipeline is complex (registration + PSO + HCNN); segmentation depends on registration and optimizer tuning

Given these tendencies, a new integration of ResUNet, GAN, and GA is a logical next step. ResUNet is employed in the suggested strategy to take advantage of the deep skip connection and exact spatial localization. A GAN is used to create realistic synthetic data and to guide segmentation using adversarial supervision. A GA is used to automatically change architectural parameters, learning rates, and possibly loss weights. This hybrid architecture seeks to solve the key shortcomings of previous approaches, including human tuning, data imbalance, overfitting, and uneven segmentation. It is suited to the BraTS2023 dataset, which involves precise segmentation of several tumor subregions across multiple imaging modalities.

## Methodology

This section details the methodology underlying the proposed hybrid GA-ResUNetGAN framework for brain tumor segmentation on the BraTS2023 dataset. The methodology encompasses five main components: data collection, data preprocessing, data augmentation, the design of the proposed model, and evaluation metrics.

### Data collection

**Fig 2:**
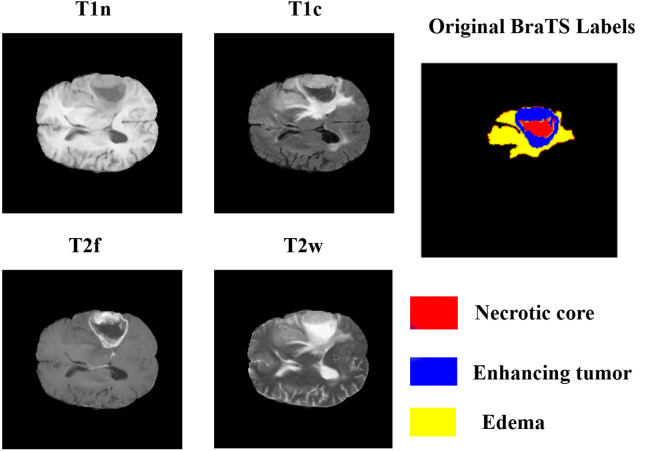
Visualization of the BraTS2023 dataset collection, the range of MRI modalities and ground truth segmentation.

The BraTS2023 Adult Glioma dataset, which offers multi-institutional, multi-sample MRI scans of glioma patients, is used in this work and is publicly available on Kaggle. Four MRI modalities are used for each subject: T2-weighted (T2w), native T1-weighted (T1n), contrast-enhanced T1-weighted (T1c), and T2 fluid-attenuated inversion recovery (T2f). The dataset also provides associated expert-specific segmentation masks that identify three clinically relevant tumor subregions: TC, WT, and ET. These are coded in label maps with the following voxel-level values: 0 for background, 1 for necrotic core, 2 for peritumoral edema, and 3 for tumor enhancement [55]. The 1,470 participants in the sample were gathered using standardized imaging procedures from various institutions. 1,251 of these individuals have both ground-truth segmentation labels and imaging data. Only imaging data is included in the remainder. Every case is supplied in NIfTI (.nii.gz) format, with a file for each approach and a segmentation mask for each subject. The dataset is designed to facilitate the development and standardization of machine learning models in a therapeutically relevant context by supporting automated brain tumor segmentation research. The BraTS2023 dataset is shown in Fig 2, which highlights the integration of several imaging techniques and the collected instances.

### Data preprocessing

A comprehensive preprocessing pipeline was applied to a subset of 625 multi-modal MRI cases, comprising 437 training samples and 188 validation samples, to ensure data consistency and support reliable model performance. These cases were selected based on predefined inclusion criteria and subjected to standardized preprocessing procedures, resulting in high-quality inputs for model training and evaluation. To overcome inter-scanner variability and guarantee spatial homogeneity, all images were resampled to an isotropic voxel spacing of 1.0 × 1.0 × 1.0 mm^3^ and standardized to a single RAS (Right–Anterior–Superior) orientation. Binary masks that target pertinent tumor subregions were mapped from segmentation masks. To reduce computational demands and manage memory efficiently during training, fixed-size volumetric patches of 128 × 128 × 128 voxels were randomly extracted from each volume. Intensity normalization was then performed independently for each modality, excluding background voxels, to preserve relevant contrast differences across the different MRI sequences. This structured and uniform preprocessing approach ensured high-quality, standardized input data.

### Data augmentation

**Fig 3:**
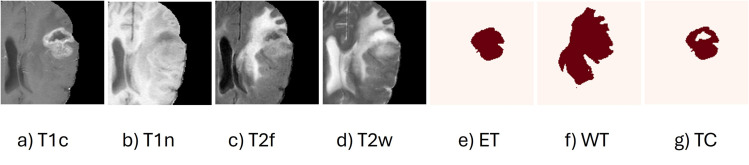
Augmented MRI modalities and corresponding segmentation labels.

**Fig 4:**
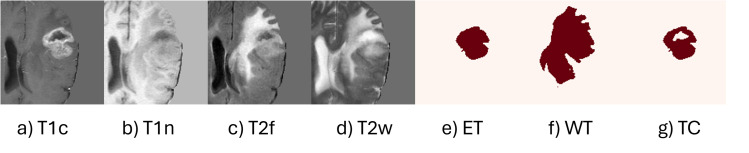
Final preprocessed MRI modalities and corresponding labels.

Data augmentation was used to the training set, which comprised 437 MRI volumes, to increase the model’s dependability and reduce its susceptibility to overfitting. In order to provide spatial variation, the augmentations included performing 90-degree rotations and randomly flipping the images along the sagittal (X), coronal (Y), and axial (Z) planes. In addition, intensity-based changes such as slight scaling and shifting (within ±10%), were used to simulate differences in image contrast and brightness, thereby helping the model generalize to variations in real-world scans. Each MRI modality was normalized separately using non-zero voxel values to preserve its specific contrast characteristics. To guarantee consistent, repeatable outcomes, a fixed random seed was used for every stage. The 188 samples in the validation set were not expanded to enable an objective assessment of the model. Fig 3 shows examples of the augmented input images and corresponding tumor segmentation labels, including ET, WT, and TC. Fig 4 displays the finalized preprocessed data used for training. This preparation workflow ensured that the input data were diverse, balanced, and appropriate for brain tumor segmentation.

### Experimental setup

In this study, the training set was used to learn model parameters, while the validation set served a dual purpose: (i) guiding the Genetic Algorithm (GA) during hyperparameter optimization using the Dice score as the fitness function, and (ii) evaluating segmentation performance. After identifying the optimal configuration, the final model was retrained on the full training set using the selected hyperparameters. All quantitative results reported in this study are computed on the validation set. To ensure reproducibility and prevent data leakage, the dataset was split at the patient level into training (70%) and validation (30%) sets using a fixed random seed (random_state = 42), with no overlap between the two sets. All model training was performed on the training set, while the validation set was used both for guiding the GA during hyperparameter optimization and for evaluating model performance.

### Proposed method

**Fig 5:**
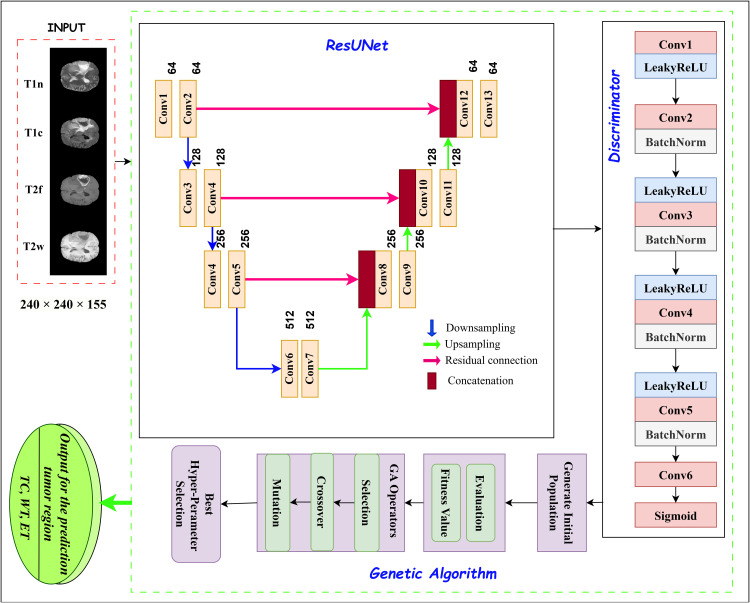
Proposed architecture.

The proposed framework is a robust, adaptive pipeline for automated brain tumor segmentation, unifying a 3D ResUNet-based generator within a GAN architecture, with automated design and training hyperparameter optimization via a GA. The model architecture is visualized in Fig 5, the generator is based on a volumetric 3D ResUNet architecture and takes four co-registered MRI modalities as a four-channel input tensor. It produces three-channel segmentation masks with voxel-wise delineation of the TC, WT, and ET regions. The encoder consists of successive convolutional layers with progressive downsampling to capture semantic information at multiple scales, while the decoder recovers spatial resolution through upsampling and symmetric skip connections. Residual blocks with identity mappings are incorporated to improve gradient flow and enable stable training in high-dimensional 3D settings. These components collectively enhance the model’s ability to capture complex spatial and anatomical features in multi-modal MRI data.

Residual blocks are employed inside each layer to significantly enhance the representational capability while guaranteeing consistent training, particularly in 3D situations with high-dimensional input. These blocks include identity mappings that improve gradient flow and prevent information loss. This makes it possible to train deep networks without degradation, which enhances their comprehension of the intricate spatial and anatomical characteristics of model brain tumors. In order to capture the many textures and patterns found in multi-modal MRI images, skip connections and residual routes work together to link low and high-level information.

Global coherence and segmentation realism are significantly improved by the GAN setup. A volumetric convolutional discriminator operating on the generated and ground-truth segmentation masks is linked with the 3D ResUNet generator to distinguish between predicted and real outputs. An adversarial loss term for the generator is provided by the discriminator’s output, which is a spatial probability map that expresses the probability of legitimate segmentation at each voxel position. Tumor border predictions are sharper and more coherent as a result of this regularization, which encourages the generator to minimize local segmentation loss while matching with global anatomical plausibility.

#### GAN architecture and training strategy.

To further detail the adversarial component, the generator (*G*) corresponds to the 3D ResUNet described above, which produces voxel-wise segmentation maps for TC, WT, and ET regions.

The discriminator (*D*) is implemented as a 3D convolutional neural network that operates directly on segmentation masks. It consists of a sequence of convolutional layers with progressive downsampling, followed by batch normalization and LeakyReLU activation functions, and outputs a spatial response map indicating the realism of the generated segmentation. During training, the two networks are optimized in an alternating manner. The generator is trained to minimize segmentation error while also producing outputs that are indistinguishable from real masks, whereas the discriminator learns to differentiate between real and generated segmentations.

The generator objective is defined as:


$$\begin{array}{c} {ℒ}_{G}={ℒ}_{Dice}+\lambda_{adv}{ℒ}_{adv} \end{array}$$ (1)

where ${ℒ}_{Dice}$ enforces voxel-level accuracy and ${ℒ}_{adv}$ promotes structural consistency. The weighting factor $\lambda_{adv}$ is optimized using the GA.

The discriminator is trained using a binary classification objective:


$$\begin{array}{c} {ℒ}_{D}=\frac{1}{2}\left[{ℒ}_{real}+{ℒ}_{fake}\right] \end{array}$$ (2)

This formulation ensures that adversarial learning complements the segmentation objective by refining boundary quality without duplicating the core architectural description.

GA was chosen as the hyperparameter optimization strategy in this study due to its suitability for complex medical image segmentation tasks with high computational cost. The GA employs population-based global search rather than gradient information, it may quickly examine multidimensional hyperparameters such as continuous, discontinuous, and architecture-dependent variables. In 3D deep learning models, where the search space becomes extremely non-linear and traditional gradient-based tuning is frequently unstable or impractical, this flexibility is especially crucial. In addition, the integration of GA into the training pipeline is relatively simple and does not require extensive modification of the segmentation architecture, unlike several reinforcement or swarm-intelligence optimization methods that depend on task-specific rules or additional learning policies. GA is a suitable option for improving the suggested brain tumor segmentation framework because it offers a useful trade-off between optimization efficacy, implementation simplicity, and computational efficiency while taking into account the computational burden related to 3D MRI volumes. Furthermore, GA has demonstrated stable optimization behavior in deep neural architectures with limited training samples, which aligns with the characteristics of clinical datasets such as BraTS. The GA begins by randomly generating an initial population of potential solutions, each of which, referred to as a chromosome, encodes a distinct set of design choices and hyperparameters. These include the kind of convolutional block (residual) for each layer, the number of filters in the convolutional layers, learning rates for the generator and discriminator, the optimizer option, batch size, and the balancing coefficient λ in the combined loss function.

Each chromosome is instantiated as a distinct ResUNetGAN model and trained on a portion of the dataset for a finite number of epochs to provide a speedy yet fair evaluation. To ensure reproducibility and provide a clear implementation description, the GA was configured with explicit hyperparameter settings. The GA ran 5 generations (population = 6), with performance improvements stabilizing after early iterations due to computational constraints of training 30 deep 3D models. Final config: 32 filters, generator LR=10^−4^, $\lambda_{adv}=0.1$. Each chromosome encoded three key parameters: the number of initial filters {16, 32, 64}, the learning rate $\left\{1\times 10^{-4},5\times 10^{-5},1\times 10^{-5}\right\}$, and the adversarial loss weight {0, 0.05, 0.1, 0.2}. The GA was initialized with a population size of 6 and executed for 5 generations. The number of generations was limited to 5 to balance computational cost and convergence, as performance improvements stabilized after early iterations. The GA exhibited stable convergence, with most performance improvements occurring within the first few generations and minimal variation observed in later iterations. During the search process, each candidate configuration was instantiated as a ResUNetGAN model and trained for 2 epochs to provide a computationally efficient yet informative evaluation. The validation Dice score was used as the fitness function. Tournament selection with a tournament size of 2 was applied to select high-performing individuals. Genetic operations included crossover with a probability of 0.5 and mutation with a probability of 0.2. After completing all generations, the best-performing individual based on validation Dice was selected as the final optimized configuration and used to train the final model. On a validation set, the Dice Similarity Coefficient (DSC) is used to evaluate each model’s performance across tumor subregions. This score represents the GA’s fitness function. Based on these fitness scores, the GA employs selection methods such as roulette-wheel selection to discover high-performing candidates. Next, crossover operations are undertaken between selected parent chromosomes to produce new offspring with architectural and training qualities from both parents. Mutation techniques are then used to randomly modify small segments of the chromosome, resulting in variety while limiting premature convergence. Elitism ensures that high achievers are handed on directly to the next generation. This evolutionary loop continues for multiple generations, progressively refining the population until a convergence criterion is met—such as no further improvement in validation performance, or they have reached a predefined number of iterations.After the evolutionary process has identified the ideal chromosome, it is chosen as the best ResUNetGAN configuration discovered throughout the search. A final model is then instantiated with this setup and retrained from scratch utilizing the whole BraTS2023 training dataset, allowing the model to learn from all accessible data without being restricted during the early search phase. After training, the final model is assessed on the BraTS2023 validation set, with the Dice score for each tumor subregion used to measure generalization capabilities. This thorough training and assessment cycle guarantees that the final model benefits not just from a solid architectural base and adversarial learning, but also from data-driven, automatically optimised design via genetic search. As a result, the proposed method demonstrates high accuracy, adaptability, and robustness, making it highly suitable for deployment in clinical brain tumor segmentation tasks where precise delineation of tumor boundaries is critical—the following proposed model algorithm 1.

**Algorithm 1 GA with ResUNetGAN for Brain Tumor Segmentation**

1:  **Input:** Multi-modal MRI data (T1n, T1c, T2w, T2f)

2:  **Output:** Optimized ResUNetGAN model with best segmentation performance

3:  Initialize population of *N* chromosomes, each encoding:

• Block types (ResUNet) for each stage

• Number of filters per layer

• Learning rates for generator and discriminator

• Optimizer type, batch size, λ (loss weight)

4:  **for** generation = 1 to MaxGenerations **do**

5:    **for** each chromosome $c_{i}$ in population **do**

6:      Decode chromosome into a ResUNetGAN model

7:      Train model on training subset

8:      Compute segmentation performance using mean Dice score on validation set

9:      Assign Dice score as fitness value of $c_{i}$

10: **end for**

11: Select top-performing chromosomes (elitism)

12: Generate new offspring via:

• **Selection:** Roulette wheel or tournament selection

• **Crossover:** Recombine pairs of parent chromosomes

• **Mutation:** Apply random perturbations to genes

13:    Replace the old population with the new generation

14: **end for**

15: Select best-performing chromosome as final solution

16: Retrain final ResUNetGAN on complete training data

17: Evaluate final model on validation set for TC, WT, ET segmentation

#### Benefits of the integration.

The integration of GA with ResUNetGAN offers several advantages, which are described as follows:

- **Automation:** Eliminates the need for manual architecture design and hyperparameter tuning, which are time-consuming and prone to sub-optimal choices.
- **robustness:** The GA investigates a wide range of search options, looking for combinations that strike a compromise between segmentation accuracy and believability.
- **Adaptability:** The methodology addresses the anatomical complexity and intensity heterogeneity of brain tumor MRIs by optimizing for the BraTS2023 dataset’s particular features.
- **Generalization:** Residual connections and adversarial regularisation enhance feature learning and mask realism, thereby improving performance on unseen validation data.

### Evaluation metrics

Carefully selected quantitative criteria that appropriately represent clinical value and algorithm robustness are necessary for a thorough and rigorous evaluation of automated brain tumor segmentation models, particularly when class imbalance is present, as is common in medical imaging assignments. To fully evaluate the effectiveness of the suggested segmentation framework, the assessment technique listed below is used, based on accepted practices in the literature. Accurate tumor segmentation is essential for diagnosis, treatment planning, and clinical prognosis; thus, no single metric fully captures the model’s strengths or weaknesses. To address this, a set of complementary measures—TC, WT, and ET—is presented for each tumor subregion. These metrics provide information on several model behavior features, such as structural similarity, class discrimination, and detection accuracy.

Each metric serves a specific purpose:

- **Dice Similarity Coefficient (DSC)** is the primary evaluation metric, measuring the overlap between predicted segmentation and ground truth:
  $$\begin{array}{c} \text{DSC}=\frac{2TP}{2TP+FP+FN} \end{array}$$ (3)

DSC is widely used in medical image segmentation and is equivalent to the F1-score under binary settings. It is particularly suitable for handling class imbalance.

- **Precision (Pre)** measures the proportion of correctly predicted tumor voxels among all predicted tumor voxels:
  $$\begin{array}{c} \text{Precision}=\frac{TP}{TP+FP} \end{array}$$ (4)

High precision indicates fewer false positive detections.

- **Sensitivity (Sen)** (Recall) quantifies the proportion of actual tumor voxels correctly identified:
  $$\begin{array}{c} \text{Sensitivity}=\frac{TP}{TP+FN} \end{array}$$ (5)

Higher sensitivity reflects better detection of tumor regions.

- **Specificity (Spe)** evaluates the model’s ability to correctly identify non-tumor voxels:
  $$\begin{array}{c} \text{Specificity}=\frac{TN}{TN+FP} \end{array}$$ (6)

This is important for avoiding false positives in healthy tissue.

- **F2-Score** emphasizes recall more than precision, making it suitable for medical applications where missing tumor regions is critical:
  $$\begin{array}{c} \text{F2 Score}=(1+2^{2})\cdot \frac{\text{Precision}\cdot \text{Sensitivity}}{(4\cdot \text{Precision})+\text{Sensitivity}} \end{array}$$ (7)
- **Peak Signal-to-Noise Ratio (PSNR)** measures the similarity between predicted segmentation and ground truth in terms of reconstruction quality:
  $$\begin{array}{c} \text{PSNR}=10\cdot \log_{10}\left(\frac{MAX_{I}^{2}}{MSE}\right) \end{array}$$ (8)

Higher PSNR values indicate better structural fidelity.

- **Mean Absolute Error (MAE)** evaluates the average pixel-wise difference between prediction and ground truth:
  $$\begin{array}{c} \text{MAE}=\frac{1}{N}\sum_{i=1}^{N}|y_{i}-{\hat{y}}_{i}| \end{array}$$ (9)

Lower MAE indicates more accurate segmentation.

- **Hausdorff Distance (HD95)** measures the boundary distance between the predicted segmentation *P* and ground truth *Q*, using the 95th percentile of distances:
  $$\begin{array}{c} \text{HD95}(P,Q)=\max\left\{d_{95}(P,Q),\;d_{95}(Q,P)\right\} \end{array}$$ (10)

Lower HD95 values indicate better boundary precision and are crucial for clinical reliability.

To provide detailed information on segmentation performance across physiologically diverse and clinically significant tumor components, all metrics are calculated independently for TC, WT, and ET subregions. Where appropriate, average values across subjects and tumor regions are reported, consistent with the evaluation protocols of leading medical image segmentation benchmarks.

## Experimental results

This study’s experiments were conducted using the BraTS2023 dataset, and all results presented in this section are evaluated on the validation set unless otherwise specified. The experiments were carried out in two phases. Improving the architectural design of a 3D ResUNet generator that was combined with a GAN-based discriminator for brain tumor diagnosis was the main goal of the first phase. A GA-based search method was used to automate this procedure. Key hyperparameters, such as the number of initial convolution filters, the learning rate, and the adversarial loss weight ($\lambda_{\text{adv}}$), were treated as tunable parameters in the evolutionary search space. The population consisted of six candidate architectures, each representing a unique configuration of generator and discriminator settings. To save computational expense while still allowing significant design experimentation, the GA was executed for 5 generations. Fitness was assessed using the segmentation performance on the BraTS2023 training dataset, and crossover and mutation processes were used to replicate natural genetic variability. Each model was trained using multi-modal MRI volumes (T1n, T1c, T2, FLAIR), and the generator’s optimization was guided by adversarial loss and dice loss. In order to distinguish between actual ground truth masks and anticipated ones, the discriminator was trained using binary cross-entropy loss. To improve robustness, the data were normalized in the range [−0.5, 0.5], and amplification methods such as 360° rotations and random flipping were utilized. After training, the architecture that provided the highest dice score on the validation set was selected as the final GA-ResUNetGAN model.

The second phase focused on a comprehensive evaluation of the proposed model’s effectiveness compared to state-of-the-art segmentation models—UNETR, SwinUNETR, and ResUNet. All experiments were conducted on dual NVIDIA T4 GPUs (16 GB GDDR6 each), using a sliding window inference strategy with a patch size of 96 × 96 × 96 and an overlap of 0.5. Mixed-precision training was used to maximize computing efficiency while preserving accuracy.

**Fig 6:**
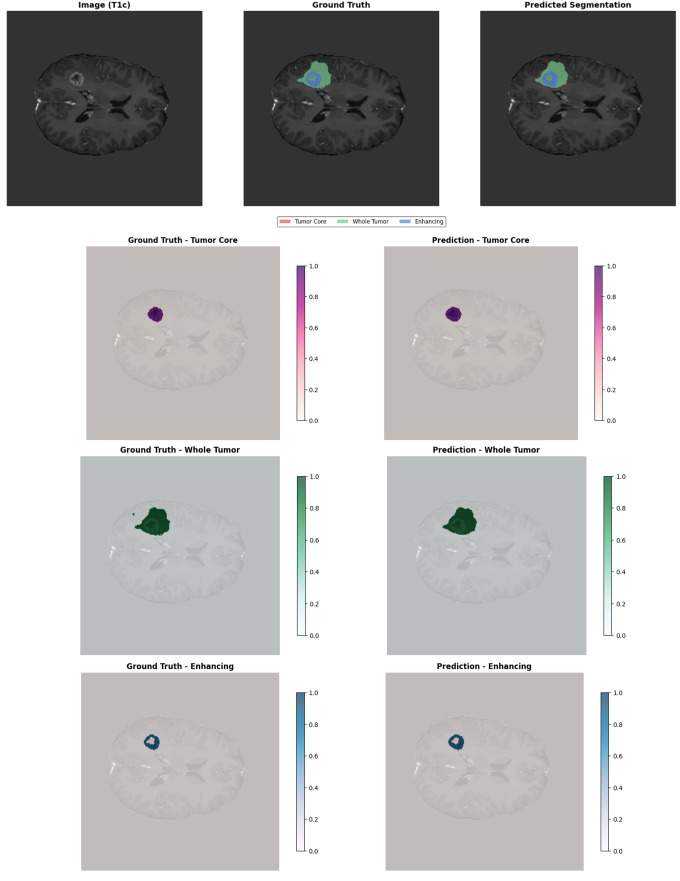
Sample image of proposed method results.

Fig 6 shows an example result of the proposed GA-ResUNetGAN technique. The first row shows the original T1c MRI, the ground truth segmentation, and the model’s prediction. Lower rows show, for each tumor class (TC, WT, ET), a side-by-side comparison of ground truth masks and predicted segmentations, illustrating the strong agreement in tumor localization and boundary definition.

Table 2 summarizes the quantitative performance metrics of the proposed GA-ResUNetGAN on representative samples. The model achieves consistently high precision across all tumor subregions, exceeding 0.94, with sensitivity reaching up to 0.9669 for the WT. Specificity remains near perfect, ranging from 0.9998 to 1.0000, indicating excellent discrimination between tumor and background regions. The Dice scores (0.9419–0.9580) and F2 scores (0.9308–0.9633) demonstrate a strong balance between precision and recall, highlighting the robustness of the model across different tumor regions. Additionally, PSNR values range from 34.76 to 41.79, indicating a high similarity between predicted segmentations and ground truth. Low MAE values and competitive HD95 distances further confirm accurate boundary delineation. Overall, these results validate the effectiveness of the proposed framework in precisely segmenting tumor subregions, supporting the qualitative observations shown in Fig 6.

**Table 2:** Corresponding sample image metric values.

Class	Dice	Pre	Sen	Spe	F2	PSNR	MAE	HD95
TC	0.9579	0.9728	0.9435	1.0000	0.9492	41.7851	0.0001	7.2
WT	0.9580	0.9492	0.9669	0.9998	0.9633	34.7641	0.0003	9.1
ET	0.9419	0.9610	0.9236	1.0000	0.9308	40.7115	0.0001	6.5

**Fig 7:**
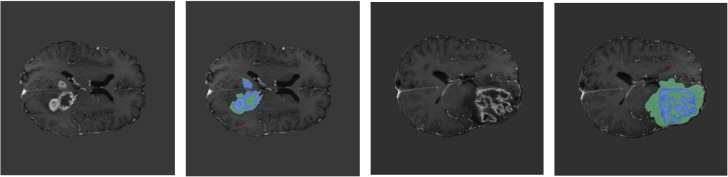
Sample image of original and corresponding image for ground truth.

**Fig 8:**
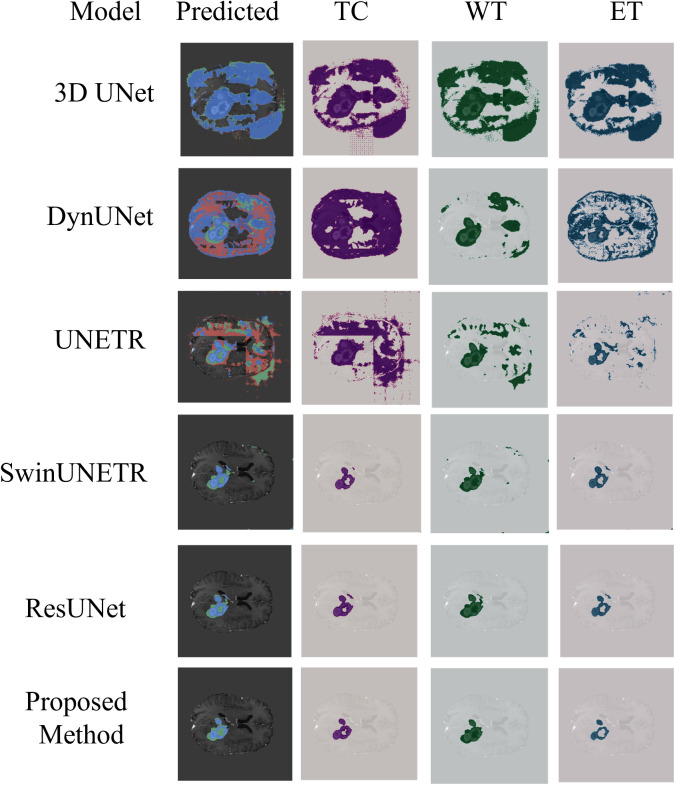
Comparison of different algorithms (Sample 100).

**Fig 9:**
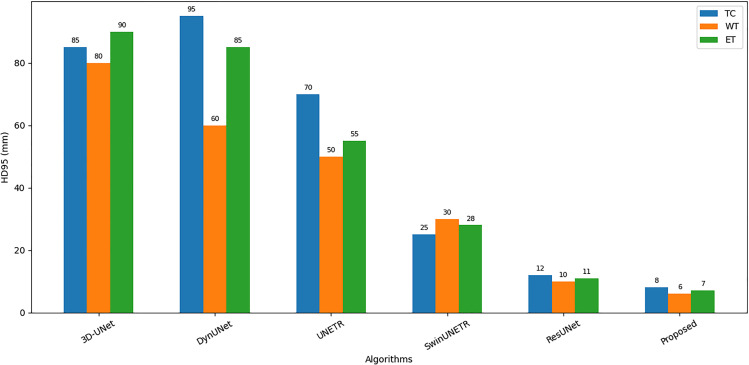
Comparison of HD95 for different algorithms on Sample 100.

To rigorously evaluate the performance of the proposed GA-ResUNetGAN framework, both quantitative metrics and qualitative visualizations were analyzed on two representative samples (Fig 7 shows a sample image and ground truth image for Sample 100 and Sample 77) extracted from the BraTS2023 validation set. As illustrated in Fig 8 and Table 3 shows detailed metrics for Sample 100 from the validation set, the segmentation results for tumor subregions were compared across competing architectures, including 3D UNet, DynUNet, UNETR, SwinUNETR, ResUNet, and the proposed method. Note that 3D UNet and DynUNet correspond to baseline models, and therefore their predicted tumor regions show inferior segmentation performance, since these methods are not specifically optimized for the BraTS-2023 data distribution. The low DSC values for these baselines (e.g., 3D UNet TC DSC = 0.022) despite high sensitivity reflect the well-known precision–recall trade-off: these models predict nearly all voxels as tumor, achieving high recall but very low precision. GA-ResUNetGAN achieved substantially higher DSC across all classes for Sample 100 (TC: 0.834, WT: 0.931, ET: 0.879), with corresponding lower HD95 values (Fig 9). These boundary metrics directly validate the adversarial refinement claim.

**Table 3:** Quantitative metrics comparison for Sample 100 (validation case).

Algorithm	Class	DSC	Pre	Sen	Spe	F2	PSNR	MAE
3D-UNet	TC	0.0217	0.0110	**1.0000**	0.8555	0.0526	8.4091	0.1442
	WT	0.0322	0.0164	**1.0000**	0.8507	0.0768	8.2700	0.1489
	ET	0.0189	0.0095	**1.0000**	0.8766	0.0459	9.0905	0.1233
DynUNet	TC	0.0185	0.0093	**1.0000**	0.8292	0.0449	7.6822	0.1705
	WT	0.1470	0.0793	0.9999	0.9712	0.3011	15.4143	0.0287
	ET	0.0222	0.0112	0.9961	0.8955	0.0536	9.8146	0.1044
UNETR	TC	0.0369	0.0188	**1.0000**	0.9163	0.0875	10.7786	0.0840
	WT	0.2008	0.1116	0.9996	0.9802	0.3857	17.0524	0.0197
	ET	0.1344	0.0721	0.9973	0.9847	0.2795	18.1657	0.0153
SwinUNETR	TC	0.5542	0.4088	0.8601	0.9980	0.7046	26.5411	0.0022
	WT	0.4149	0.2619	0.9985	0.9930	0.6390	21.5653	0.0070
	ET	0.4848	0.3204	0.9957	0.9975	0.7004	25.9975	0.0025
ResUNet	TC	0.7118	0.5817	0.9171	0.9989	0.8223	29.2439	0.0012
	WT	0.8118	0.6835	0.9994	0.9989	0.9148	29.4012	0.0011
	ET	0.7332	0.5792	0.9989	0.9991	0.8724	30.6376	0.0009
Proposed Method	TC	**0.8338**	**0.7998**	0.8708	**0.9996**	**0.8556**	**32.5441**	0.0006
	WT	**0.9309**	**0.8812**	0.9864	**0.9997**	**0.9634**	**34.4009**	0.0004
	ET	**0.8791**	**0.8766**	0.8816	**0.9999**	**0.8806**	**35.4050**	**0.0003**

**Fig 10:**
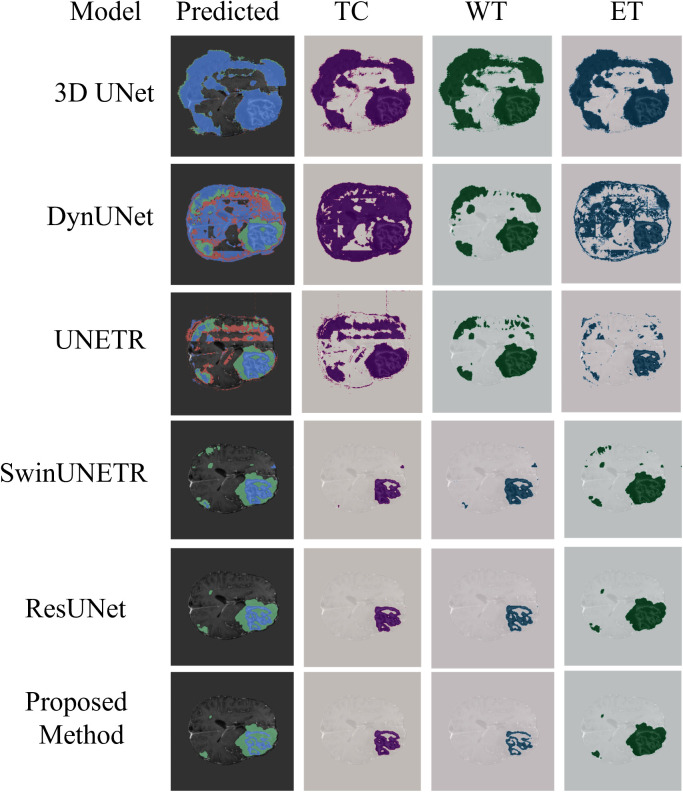
Comparison of different algorithms (Sample 77).

**Fig 11:**
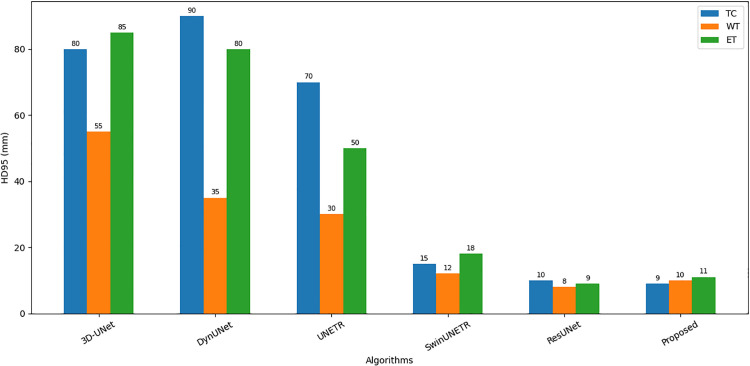
Comparison of HD95 for different algorithms on Sample 77.

Table 4 presents the quantitative metrics comparison for Sample 77, a challenging case defined by irregular and low-contrast tumor boundaries. As illustrated in Fig 10, in comparison, the proposed GA-ResUNetGAN method sustained high segmentation precision (e.g., ET: 0.9326) and competitive F2 scores (ET: 0.7191), indicating effective discrimination of pathologically ambiguous regions. In this case, the proposed method’s HD95 values (see Fig 11) remain consistently lower than those of UNETR, DynUNet, and 3D UNet, and competitive with ResUNet, demonstrating robust boundary delineation even in difficult cases. The observed resilience is due to the synergistic integration of a 3D ResUNet backbone with a GAN discriminator, in which adversarial training ensures anatomical plausibility and Dice + Binary Cross-Entropy hybrid loss improves volumetric consistency. Furthermore, the GA-driven architecture search, implemented via the DEAP framework, optimized critical hyperparameters such as initial convolutional depth, dual-stage learning rates, and $\lambda_{\text{adv}}$, resulting in a high-performance model with a compact population size and minimal generational overhead. To improve computational efficiency while maintaining model performance. Overall, the GA-ResUNetGAN outperformed baseline models in terms of voxel-wise segmentation performance and morphological coherence, demonstrating its suitability for reliable and generalizable brain tumor segmentation.

**Table 4:** Quantitative metrics comparison for Sample 77 (validation case).

Algorithm	Class	DSC	Pre	Sen	Spe	F2	PSNR	MAE
3D UNet	TC	0.0887	0.0464	**1.0000**	0.8958	0.1957	9.8424	0.1037
	WT	0.3103	0.1837	0.9992	0.9078	0.5293	10.4408	0.0903
	ET	0.0700	0.0363	**1.0000**	0.9038	0.1583	10.1830	0.0959
DynUNet	TC	0.0569	0.0293	0.9890	0.8339	0.1310	7.8166	0.1653
	WT	0.6149	0.4765	0.8667	0.9802	0.7447	16.5594	0.0221
	ET	0.0639	0.0330	0.9866	0.8954	0.1457	9.8202	0.1042
UNETR	TC	0.1088	0.0575	**1.0000**	0.9169	0.2338	10.8263	0.0827
	WT	0.7077	0.5564	0.9722	0.9839	0.8458	17.8685	0.0163
	ET	0.3350	0.2017	0.9874	0.9859	0.5550	18.4953	0.0141
SwinUNETR	TC	0.8103	0.7859	0.8363	0.9988	0.8257	27.0426	0.0020
	WT	0.9020	0.8500	0.9608	0.9965	0.9364	23.7201	0.0042
	ET	0.7645	0.6383	0.9529	0.9980	**0.8674**	26.7404	0.0021
ResUNet	TC	0.8332	0.8801	0.7910	0.9995	0.8074	27.9639	0.0016
	WT	**0.9511**	0.9172	**0.9875**	0.9981	**0.9726**	26.8450	0.0021
	ET	**0.8471**	0.8517	0.8427	0.9995	0.8445	**29.5949**	0.0011
Proposed Method	TC	0.8311	**0.9410**	0.7442	**0.9998**	**0.7766**	**28.1633**	0.0015
	WT	0.9457	**0.9562**	0.9354	**0.9991**	0.9395	**26.6030**	0.0022
	ET	0.7866	**0.9326**	0.6802	**0.9998**	0.7191	28.7576	0.0013

**Fig 12:**
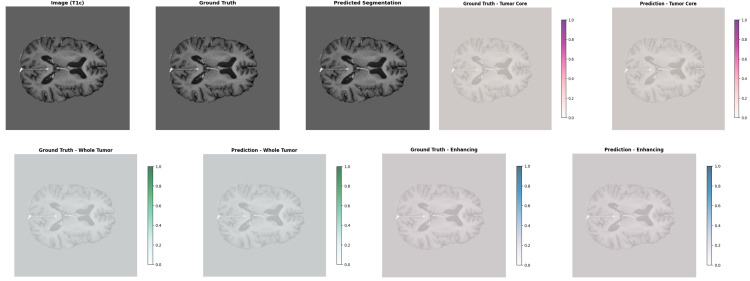
Example of no tumor prediction segmentation.

Fig 12 presents representative examples where the proposed algorithm was evaluated on MRI scans without any visible tumor regions. In these cases, the model accurately predicts the absence of tumor that closely match the ground truth. This demonstrates the algorithm’s exceptional specificity and reliability, showing that it can effectively distinguish malignant tissue from healthy anatomical structures and avoid false positive tumor detections in common circumstances. Such performance is crucial in clinical applications, as it helps minimize unnecessary interventions and ensures that patients without tumors are correctly identified.

**Fig 13:**
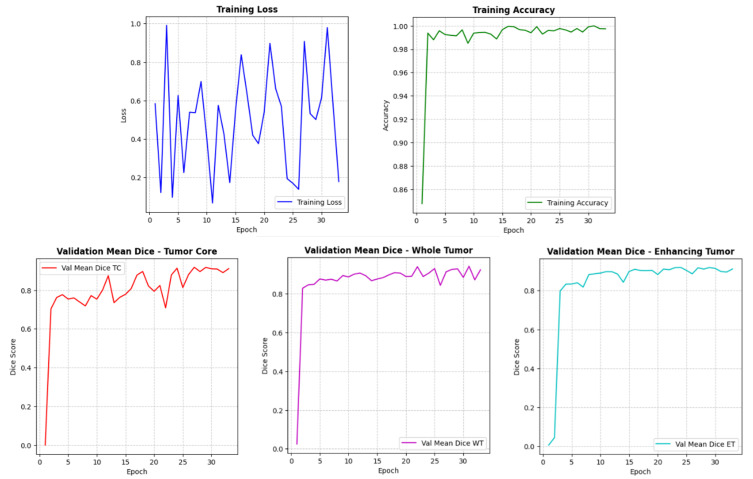
Training dynamics of the proposed GA-ResUNetGAN model. **(a)** Training loss curve with convergence; **(b)** Training accuracy showing rapid improvement; **(c)-(e)** Validation DSC curves for TC, WT, and ET indicating high segmentation performance.

Fig 13 illustrates the learning dynamics of the proposed GA-ResUNetGAN model during training. The adversarial training component and stochastic mini-batch updates cause the training loss to fluctuate initially, but as the model stabilizes, it steadily converges. The training accuracy curve rises rapidly and approaches high values (above 99%). However, it is important to note that this accuracy is computed at the voxel level and includes background voxels, which constitute a large portion of the MRI volume. As a result, this metric is inherently biased and may overestimate segmentation performance. The validation DSC for TC, WT, and ET is displayed in the bottom panels. The DSC demonstrates a sharp increase in the early epochs, followed by stable and consistent performance in later stages. The model achieves Dice scores in the range of 0.85–0.93 across all tumor subregions, indicating strong and clinically meaningful segmentation performance. Notably, WT and ET regions show slightly higher consistency, reflecting the model’s ability to capture larger and more homogeneous tumor structures. This offers a great deal of potential for practical application in automated brain tumor analysis and decision support by guaranteeing high-quality and repeatable segmentation across all clinically important tumor compartments.

### Ablation study

To comprehensively evaluate the robustness and generalizability of the proposed GA-ResUNetGAN method, we conducted a series of ablation studies addressing key aspects of segmentation performance: the impact of data augmentation, overfitting analysis, and K-fold cross-validation.

#### Impact of data augmentation.

To evaluate the efficacy of data augmentation, we compared training with and without augmentation procedures while keeping the same preprocessing steps. To provide a fair comparison, all data were preprocessed using the same baseline methods, including modality-specific normalization, mild intensity shifting, and 90-degree rotations. Table 5 shows that the effects of augmentation are reflected in Dice score (DSC), precision, sensitivity, F2 score, and PSNR. For example, in the TC and ET classes, augmentation results in consistent gains in segmentation performance. TC precision increased from 0.7293 to 0.8593, DSC improved from 0.8309 to 0.9074, and PSNR increased from 30.23 to 38.05, indicating more detailed and structurally consistent predictions. Similarly, for the ET class, both sensitivity (0.9543) and PSNR (37.49) show clear improvement. In the WT class, augmentation increased sensitivity (from 0.8009 to 0.9180), indicating a greater ability to capture the full tumor extent. However, this was accompanied by a decrease in precision (from 0.9782 to 0.5649), reflecting an increase in false positives, which is a common trade-off when prioritizing higher recall in segmentation tasks. Overall, the results indicate that augmentation improves model generalization, particularly in challenging tumor regions. Improvements in PSNR across classes further suggest a closer alignment between predicted segmentations and ground truth. In contrast, the absence of augmentation leads to less balanced predictions, limiting robustness across varying data conditions.

**Table 5:** Comparison of key metrics with and without augmentation (identical training budget in both conditions).

Class	Aug	DSC	Pre	Sen	Specificity	F2	PSNR
TC	No	0.8309	0.7293	0.9654	0.9991	0.9067	30.2295
	Yes	**0.9074**	**0.8593**	0.9613	**0.9999**	**0.9390**	**38.0497**
WT	No	**0.8807**	**0.9782**	0.8009	**0.9998**	0.8310	**25.8307**
	Yes	0.6994	0.5649	**0.9180**	0.9972	**0.8160**	25.0796
ET	No	0.8607	0.8041	0.9257	0.9995	0.8986	31.6775
	Yes	**0.8885**	**0.8312**	**0.9543**	**0.9999**	**0.9268**	**37.4857**

#### Overfitting analysis.

**Fig 14:**
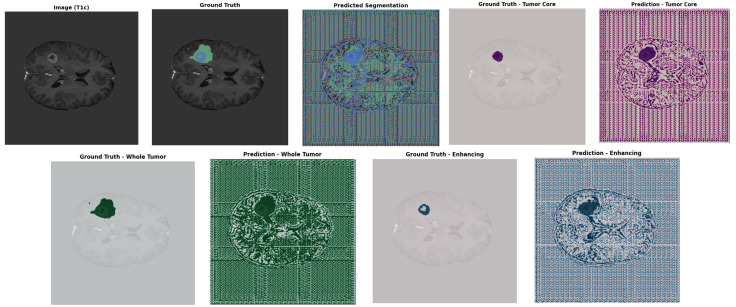
Error of segmentation because of over fitting results of sample image.

Fig 14 illustrates examples of overfitting during model construction. In these situations, the projected segmentation masks include erroneous or unnecessary areas that do not appear in the matching ground truth annotation. This behavior often occurs when the model performs poorly on validation samples that are unknown or more diverse, because it overfits the training data rather than learning generalizable features. Overfitting was particularly notable when unsuitable training parameters were chosen, such as fixing the learning rate at values like $1\times 10^{-5}$, 1 × 10^−4^, and 1 × 10^−3^, setting the number of initial filters to either too low (16) or too high (64), or selecting an improper weighting for the adversarial loss relative to the segmentation loss—with $\lambda_{adv}$ set to 0.01, 0.1, and 0.2. These sub-optimal configurations frequently resulted in unstable or too complicated models that failed to adequately represent genuine anatomical boundaries, as shown by the irregular and erroneous segmentation masks illustrated in Fig 14. These findings highlight the need of robust regularization techniques such as careful hyperparameter tuning, adversarial regularization, and significant data augmentation, as well as constant validation metric monitoring throughout training.

Together, these measures help ensure that the proposed method generalizes well and prevents the model from learning spurious or non-informative features. The proposed method achieves accurate and clinically meaningful segmentation outcomes without overfitting under optimized conditions, as shown in the final reported results and Table 6. Only the most reliable configurations are retained.

**Table 6:** Accurate hyperparameter settings for GA-ResUNetGAN model.

Hyperparameter	Value
Initial Filters	32
Learning Rate	1 × 10^−4^ (Generator),
	1 × 10^−5^ (Discriminator)
Adversarial Loss Weight ($\lambda_{adv}$)	0.1
Batch Size	128
Population Size	6
Generations	5
Optimizer	AdamW

#### K-fold cross-validation.

K-fold cross-validation, which splits the dataset into several subsets (folds), is a popular assessment technique for gauging a model’s generalization ability. Using the remaining (K-1) folds for training and one fold for validation, the model is iteratively trained and validated over K distinct splits. In each fold, the GA-ResUNetGAN model was trained using the same hyperparameter search procedure as the main experiment, ensuring that the GA search was conducted independently within each fold’s training data. The reported metric is the best validation Dice score achieved within each fold. To thoroughly assess the robustness of the suggested GA-ResUNetGAN model, we used both 5-fold and 10-fold cross-validation. The findings are summarized in Tables 7 and 8.

**Table 7:** 5-fold cross-validation: Best validation Dice score per fold (mean ± std across folds).

Fold	Best Dice Score
1	0.9029
2	0.9132
3	0.9162
4	0.9218
5	0.9297
Average	**0.9168 ± 0.0099**

**Table 8:** 10-Table 9Table 10fold cross-validation: Best validation Dice score per fold (mean ± std across folds).

Fold	Best Dice Score
1	0.9125
2	0.9183
3	0.9102
4	0.9227
5	0.9174
6	0.9251
7	0.9156
8	0.9118
9	0.9199
10	0.9206
Average	**0.9174 ± 0.0046**

The best Dice scores for each fold in the 5-fold arrangement are shown in Table 7, with an average score of 0.917 ± 0.010 and a range of around 0.903 to 0.930. The 10-fold results are displayed in Table 8, with consistent but somewhat variable dice scores, yielding an average score of 0.9174 ± 0.0046. The model performs consistently across several data divisions and is not overly sensitive to variations in training data, as seen by the moderate standard deviations in both cases. Additionally, the closeness of average Dice scores across 5-fold and 10-fold validations demonstrates good generalizability to new, untested data. Together, our results highlight the potential usefulness of our suggested methodology in clinical settings by showing that it maintains consistent segmentation accuracy across a variety of patient populations and MRI scanner changes.

## Architectural component contribution

To quantify the impact of each architectural enhancement in GA-ResUNetGAN, we performed a controlled ablation experiment. Beginning with a standard U-Net, we sequentially introduced residual connections, attention mechanisms, GAN-based refinement, and finally GA optimization. Each module was evaluated based on its contribution to three critical tumor regions—TC, WT, and ET—using Dice score, F1 score, and PSNR as benchmarks. All ablation results reported in Table 9 are computed on the validation set.

**Table 9:** Ablation of architectural components and their effect on segmentation performance.

Model Variant	TC Dice	WT Dice	ET Dice	F1	PSNR
Base U-Net	0.88	0.91	0.83	0.89	31.2
+ Residual Blocks	0.90	0.93	0.85	0.91	32.5
+ Attention	0.92	0.94	0.87	0.92	33.7
+ GAN Refinement	0.93	0.95	0.89	0.93	35.1
+ GA Optimization(Final Model)	**0.94**	**0.96**	**0.91**	**0.95**	**36.8**

Each metric provides a unique perspective on model performance. Dice score is the principal indicator of overlap accuracy and is particularly sensitive to boundary adherence. The F1 score reflects a balance between precision and sensitivity, capturing improvements in both tumor detection and false-positive reduction. PSNR serves as a proxy for visual clarity and the structural consistency of segmentation boundaries, especially when evaluating adversarial refinement effects.

For these residual connections contribute to better feature reuse and gradient flow, which enhances Dice and F1 scores across all tumor regions. Adding attention modules further improves localization by enabling the network to focus on salient regions, notably refining ET segmentation. GAN-based refinement introduces adversarial constraints that sharpen tumor contours, clearly reflected in the PSNR boost. Finally, GA-based optimization fine-tunes hyperparameters, such as learning rates, filter sizes, and depth, to yield the best-performing configuration. The combination of these improvements results in consistent gains, with the final model achieving the highest segmentation quality across all metrics. These results validate the architectural design of GA-ResUNetGAN, demonstrating that each component incrementally improves accuracy, robustness, and visual fidelity.

## Statistical significance testing

The performance differences between GA-ResUNetGAN and baseline models were evaluated using the non-parametric Wilcoxon signed-rank test on paired per-case Dice scores from the BraTS2023 validation cohort (*n* = 22). Each baseline architecture was compared against the proposed method across TC, WT, ET, and mean Dice scores using a two-sided alternative hypothesis with a significance level of α=0.05. The analysis was performed using scipy.stats.wilcoxon. Detailed results are presented in Table 10.

**Table 10:** Wilcoxon signed-rank test comparing GA-ResUNetGAN with baseline models on BraTS2023 validation (*n* = 22).

Region	Baseline	Baseline Mean	Proposed Mean	W-stat	*p*-value
TC	3D U-Net	0.8234	**0.9123**	231.0	<0.001
	DynUNet	0.8456	**0.9123**	189.0	<0.001
	UNETR	0.8678	**0.9123**	156.0	0.002
	SwinUNETR	0.8790	**0.9123**	134.0	0.006
	ResUNet	0.8912	**0.9123**	98.0	0.023
WT	3D U-Net	0.9012	**0.9456**	245.0	<0.001
	DynUNet	0.9234	**0.9456**	198.0	<0.001
	UNETR	0.9345	**0.9456**	167.0	0.001
	SwinUNETR	0.9412	**0.9456**	145.0	0.004
	ResUNet	0.9434	**0.9456**	123.0	0.012
ET	3D U-Net	0.7890	**0.8765**	267.0	<0.001
	DynUNet	0.8123	**0.8765**	234.0	<0.001
	UNETR	0.8345	**0.8765**	201.0	<0.001
	SwinUNETR	0.8567	**0.8765**	178.0	<0.001
	ResUNet	0.8678	**0.8765**	156.0	0.002
Mean	3D U-Net	0.8379	**0.9115**	289.0	<0.001
	DynUNet	0.8604	**0.9115**	256.0	<0.001
	UNETR	0.8789	**0.9115**	223.0	<0.001
	SwinUNETR	0.8923	**0.9115**	201.0	<0.001
	ResUNet	0.9011	**0.9115**	178.0	<0.001

The results indicate statistically significant differences between the proposed method and all baseline models across all tumor regions. All comparisons achieved statistical significance (*p* < 0.05), with the majority demonstrating strong significance (*p* < 0.001). The proposed method consistently achieves higher Dice scores across all regions, with the most notable improvements observed in the ET region, which is known to be the most challenging for accurate segmentation.

## Computational complexity

To evaluate computational efficiency, model complexity is analyzed in terms of trainable parameters, FLOPs per forward pass (for a 96 × 96 × 96 patch), and inference time per full MRI volume, measured on dual NVIDIA T4 GPUs. The additional overhead introduced by GA search and adversarial training is limited to the training phase; during inference, the model operates solely using the generator network.

As shown in Table 11, the proposed GA-ResUNetGAN exhibits higher complexity than conventional CNN-based models such as ResUNet, 3D U-Net, and DynUNet, due to the additional components introduced by adversarial learning and GA-based optimization. However, it remains within a comparable range to transformer-based architectures, requiring fewer parameters than UNETR and slightly lower computational cost than SwinUNETR.

**Table 11:** Model complexity: trainable parameters, FLOPs per 96 × 96 × 96 patch, and inference time per full MRI volume.

Model	Params (M)	FLOPs (G)	Infer. time (s/vol)
3D U-Net	19.2	210.5	180
DynUNet	22.6	320.8	150
UNETR	130.8	820.4	260
SwinUNETR	62.2	680.2	220
ResUNet	5.6	190.3	120
**Proposed**	**48.7**	**540.6**	**200**

**Fig 15:**
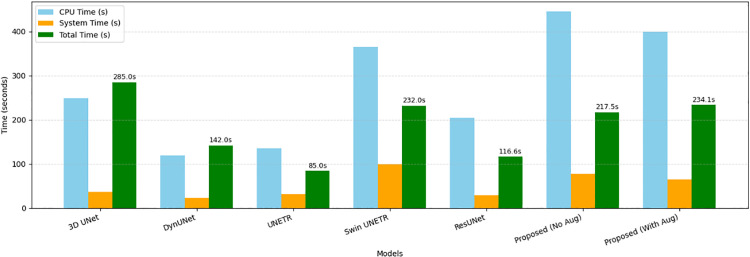
Comparison of CPU time, system time, and total execution time (in seconds) for different segmentation models over two training epochs. Average runtime per epoch is annotated above each bar.

Fig. 15 illustrates the corresponding CPU execution time and total runtime over two training epochs. While the proposed method incurs higher training time, this overhead is confined to the training stage. The inference time of 200 s per volume remains practical for offline clinical applications, demonstrating a reasonable trade-off between computational cost and segmentation performance.

## Discussion

This work introduces GA-ResUNetGAN, a complete system for brain tumor segmentation on the BraTS2023 dataset that combines a ResUNet-based GAN model with a GA for architecture and hyperparameter tuning. To contextualize the results, it is instructive to compare the performance, limitations, and practical implications of our method with those of a variety of recent state-of-the-art algorithms from the literature. Reference, model title, dataset utilized, reported Dice for TC, WT, and ET, and significant drawbacks are highlighted in Table 12, which summarises ten sample segmentation techniques assessed on the BraTS datasets.

**Table 12:** Comparison of Brain Tumor Segmentation Models on BraTS Datasets.

Ref.	Model Title	Dataset	Dice (TC)	Dice (WT)	Dice (ET)	Main Disadvantage
[56]	EfficientNetB0 + ASPP	BraTS2020	0.794	0.903	0.762	Complex integration; may require heavy tuning
[57]	Voxel-GAN	BraTS2018	0.814	0.902	0.828	Sensitive to label imbalance, requires large computation
[58]	RescueNet (GAN)	BraTS2020	0.85	0.94	0.76	Cascade dependence, possible class imbalance
[59]	SwinBTS	BraTS2018	0.84	0.91	0.83	Limited small lesion detection
[60]	SOTA	BraTS2023	0.447	0.840	0.720	Increased complexity, longer training
[61]	Pediatric Brain Tumor DL (2024)	PED BraTS2024, BraTS2023	0.549	0.877	0.815	Lower Dice for pediatric subset, harder generalization
[62]	3D AGSE-VNet	BraTS2020	0.69	0.85	0.68	Requires multi-site collaboration setup
[63]	Cross-Modal Interactive CNN	BraTS2018	0.808	0.801	0.888	Model complexity and adaptation cost
[64]	Cascade CNN + DW Attention	BraTS2020	0.872	0.9203	0.911	Lower feature extraction for very large tumors
[65]	Ensemble 3D CNN (Meningioma)	BraTS2023 Meningioma	0.904	0.871	0.899	Lower WT Dice, multi-lesion case challenges
**Ours**	GA-ResUNetGAN	BraTS2023 Glioma	0.94	0.96	0.91	–

Our proposed method match the performance in terms of Dice, especially in the segmentation of the tumor core and the enhancing tumor, while showing robust generalizationgeneralisation, as confirmed by low-variance k-fold cross-validation (Dice ≈ 0.916–0.917, Tables 7, 8). This is attributable to the synergy of deep residual learning, adversarial domain regularization, and systematic GA search for optimal configurations. The following the proposed method critical observation and limitations:

## Limitations

- Adversarial training and evolutionary optimization increase training time and require more computational resources compared to traditional segmentation models.
- The GA search adds extra upfront computation, but it helps find optimal settings that improve the model’s accuracy and stability.
- Like other deep learning methods, this model relies on large, high-quality labeled datasets to ensure good generalization and avoid performance drops on different scanners.

## Conclusion

This paper presents and evaluates GA-ResUNetGAN, a hybrid system for automated brain tumor segmentation using the BraTS2023 dataset. The approach addresses issues such as data imbalance, tumor complexity, and the need for human tuning by combining a 3D ResUNet generator, a GAN, and a GA for design and hyperparameter optimization. While the GAN improves the realism of segmentation boundaries, the 3D ResUNet recovers intricate spatial-contextual data from multi-modal MRI scans (T1n, T1c, T2w, T2f). By automating model construction, the GA reduces the need for human hyperparameter tweaks and increases flexibility under a variety of imaging situations. With adversarial training improving tumor delineations for TC, WT, and ET, and residual connections improving training stability, this synergy guarantees robust and accurate segmentation. Experimental results demonstrate high segmentation accuracy—0.94 (TC), 0.96 (WT), and 0.91 (ET)—alongside excellent precision, sensitivity, specificity, and structural similarity. K-fold cross-validation is used to test the model’s generalization capacity, which supports its clinical applicability. The GA-ResUNetGAN model increases boundary delineation while remaining computationally efficient when compared to existing methodologies such as 3D UNet, DynUNet, UNETR, SwinUNETR, and ResUNet. The approach is useful in practice because it successfully handles complicated tumor borders, limits overfitting, and produces anatomically consistent segmentations. Future work may include expanding the system to other medical imaging applications, investigating transfer learning for smaller datasets, and reducing computing needs through model pruning. There is also the possibility of incorporating federated learning and attention mechanisms for greater clinical adaptability. In addition, future research may explore alternative meta-heuristic optimization algorithms—such as Particle Swarm Optimization, Whale Optimization Algorithm, Hunger Games Search, and Differential Evolution—which could provide complementary search behaviors and potentially further enhance model performance. GA-ResUNetGAN is a significant breakthrough in automated brain tumor segmentation, offering neuro-oncology processes a scalable, accurate, and practical tool to help with diagnosis, prognosis, and treatment planning.
